# Polymorphisms within the *TNFSF4* and *MAPKAPK2* Loci Influence the Risk of Developing Invasive Aspergillosis: A Two-Stage Case Control Study in the Context of the aspBIOmics Consortium

**DOI:** 10.3390/jof7010004

**Published:** 2020-12-23

**Authors:** Jose Manuel Sánchez-Maldonado, Ana Moñiz-Díez, Rob ter Horst, Daniele Campa, Antonio José Cabrera-Serrano, Manuel Martínez-Bueno, María del Pilar Garrido-Collado, Francisca Hernández-Mohedo, Laura Fernández-Puerta, Miguel Ángel López-Nevot, Cristina Cunha, Pedro Antonio González-Sierra, Jan Springer, Michaela Lackner, Laura Alcazar-Fuoli, Luana Fianchi, José María Aguado, Livio Pagano, Elisa López-Fernández, Esther Clavero, Leonardo Potenza, Mario Luppi, Lucia Moratalla, Carlos Solano, Antonio Sampedro, Manuel Cuenca-Estrella, Cornelia Lass-Flörl, PCRAGA Study Group, Federico Canzian, Juergen Loeffler, Yang Li, Hermann Einsele, Mihai G. Netea, Lourdes Vázquez, Agostinho Carvalho, Manuel Jurado, Juan Sainz

**Affiliations:** 1 Genomic Oncology Area, GENYO, Centre for Genomics and Oncological Research, Pfizer/University of Granada/Andalusian Regional Government, PTS Granada, 18016 Granada, Spain; josemanuel.sanchez@genyo.es (J.M.S.-M.); ana.moniz@genyo.es (A.M.-D.); antoniocabreraserrano@gmail.com (A.J.C.-S.); manuel.jurado.sspa@juntadeandalucia.es (M.J.); 2 Hematology Department, Virgen de las Nieves University Hospital, 18012 Granada, Spain; mariap.garrido.sspa@juntadeandalucia.es (M.d.P.G.-C.); paquihernandez@yahoo.es (F.H.-M.); lfernandez@fibaosalud.com (L.F.-P.); pedroa.gonzalez.sspa@juntadeandalucia.es (P.A.G.-S.); eliku77@hotmail.com (E.L.-F.); eclaverosa@hotmail.com (E.C.); lucia.moratalla.sspa@juntadeandalucia.es (L.M.); 3 Instituto de Investigación Biosanitaria de Granada (ibs.GRANADA), Complejo Hospitales Universitarios de Granada/Universidad de Granada, 18016 Granada, Spain; 4 Department of Internal Medicine, Radboud Center for Infectious Diseases, Radboud University Nijmegen Medical Center, 6500HB Nijmegen, The Netherlands; RterHorst@cemm.oeaw.ac.at (R.t.H.); yangli.hunu@gmail.com (Y.L.); mihai.netea@radboudumc.nl (M.G.N.); 5 Department of Genetics, University of Pisa, 56128 Pisa, Italy; daniele.campa@unipi.it; 6 Genomic Medicine Area, GENYO, Centre for Genomics and Oncological Research, Pfizer/University of Granada/Andalusian Regional Government, PTS Granada, 18016 Granada, Spain; manuel.martinez@genyo.es; 7 Immunology Department, Virgen de las Nieves University Hospital, 18012 Granada, Spain; mavenot@ugr.es; 8 Life and Health Sciences Research Institute (ICVS), School of Medicine, University of Minho, 4710-057 Braga, Portugal; cristinacunha@med.uminho.pt (C.C.); agostinhocarvalho@med.uminho.pt (A.C.); 9 ICVS/3B’s-PT Government Associate Laboratory, 4710-057 Braga/Guimarães, Portugal; 10 Hematology Department, Universitätsklinikum Würzburg, Medizinische Klinik II, 97080 Würzburg, Germany; bkr@goldmail.de (J.S.); Loeffler_J@ukw.de (J.L.); Einsele_H@ukw.de (H.E.); 11 Division of Hygiene and Medical Microbiology, Medical University of Innsbruck, 6020 Innsbruck, Austria; Michaela.lackner@i-med.ac.at (M.L.); cornelia.lass-floerl@i-med.ac.at (C.L.-F.); 12 Mycology Reference Laboratory, Centro Nacional de Microbiología, Instituto de Salud Carlos III, 28222 Madrid, Spain; lalcazar@isciii.es (L.A.-F.); mcuenca-estrella@isciii.es (M.C.-E.); 13 Istituto di Ematologia, Università Cattolica del S. Cuore, 00168 Rome, Italy; luana.fianchi@policlinicogemelli.it (L.F.); livio.pagano@policlinicogemelli.it (L.P.); 14 Unit of Infectious Diseases, Hospital Universitario “12 de Octubre”, Instituto de Investigación Hospital “12 de Octubre” (i+12), 28041 Madrid, Spain; jaguadog1@gmail.com; 15 Department of Medical and Surgical Sciences, University of Modena and Reggio Emilia, AOU Policlinico, 41125 Modena, Italy; leonardo.potenza@unimore.it (L.P.); mario.luppi@unimore.it (M.L.); 16 Hematology Department, Hospital Clínico Universitario-INCLIVA, University of Valencia, 46010 Valencia, Spain; carlos.solano@uv.es; 17 Department of Microbiology, Virgen de las Nieves University Hospital, 18012 Granada, Spain; antonio.sampedro.sspa@juntadeandalucia.es; 18 Polymerase Chain Reaction/Aspergillus Galactomannan Antigen Study Group (PCRAGA Study Group), 37007 Salamanca, Spain; juan.sainz@gmail.com; 19 Genomic Epidemiology Group, German Cancer Research Center (DKFZ), 69120 Heidelberg, Germany; f.canzian@dkfz-heidelberg.de; 20 Centre for Individualised Infection Medicine (CiiM) & TWINCORE, Joint Ventures between the Helmholtz-Centre for Infection Research (HZI) and the Hannover Medical School (MHH), 30625 Hannover, Germany; 21 Department for Immunology & Metabolism, Life and Medical Sciences Institute (LIMES), University of Bonn, 53115 Bonn, Germany; 22 Hematology Department, University Hospital of Salamanca, 37007 Salamanca, Spain; lvazlo@usal.es; 23 Department of Medicine, University of Granada, 18016 Granada, Spain

**Keywords:** invasive aspergillosis, *TNFSF4*, *MAPKAPK2*, genetic susceptibility, B cells, monocytes, serum biomarkers, TSLP, TNFSF14

## Abstract

Here, we assessed whether 36 single nucleotide polymorphisms (SNPs) within the *TNFSF4* and *MAPKAPK2* loci influence the risk of developing invasive aspergillosis (IA). We conducted a two-stage case control study including 911 high-risk patients diagnosed with hematological malignancies that were ascertained through the aspBIOmics consortium. The meta-analysis of the discovery and replication populations revealed that carriers of the *TNFSF4*_rs7526628T/T_ genotype had a significantly increased risk of developing IA (*p* = 0.00022). We also found that carriers of the *TNFSF4*_rs7526628T_ allele showed decreased serum levels of TNFSF14 protein (*p* = 0.0027), and that their macrophages had a decreased fungicidal activity (*p* = 0.048). In addition, we observed that each copy of the *MAPKAPK2*_rs12137965G_ allele increased the risk of IA by 60% (*p* = 0.0017), whereas each copy of the *MAPKAPK2*_rs17013271T_ allele was estimated to decrease the risk of developing the disease (*p* = 0.0029). Mechanistically, we found that carriers of the risk *MAPKAPK2*_rs12137965G_ allele showed increased numbers of CD38+IgM-IgD- plasmablasts in blood (*p* = 0.00086), whereas those harboring two copies of the allele had decreased serum concentrations of thymic stromal lymphopoietin (*p* = 0.00097). Finally, we also found that carriers of the protective *MAPKAPK2*_rs17013271T_ allele had decreased numbers of CD27-IgM-IgD- B cells (*p* = 0.00087) and significantly lower numbers of CD14+ and CD14+CD16- cells (*p* = 0.00018 and 0.00023). Altogether, these results suggest a role of the *TNFSF4 and MAPKAPK2* genes in determining IA risk.

## 1. Introduction

Invasive aspergillosis (IA) is the second-most common opportunistic mycosis, and frequently affects allogenic stem cell and solid transplantation hosts [1], but also hematological patients receiving myeloablative therapies and critically ill patients [2,3]. Epidemiological studies have demonstrated that, despite the advent of new antifungal agents, IA incidence remains unacceptably high and it is associated with poor prognosis [4]. Although the molecular mechanisms underlying host defense against invasive fungal infections have been extensively studied during recent years [5], the etiopathogenesis of this opportunistic infection is still poorly understood, which hampers the development of new and effective therapeutic strategies. A large number of candidate gene association studies developed over the last decade have also shown that genetic determinants influence both the susceptibility and outcome of the infection [6]. However, most of these studies have been focused on pathogen-recognition receptors (PRRs) (*PTX3*, *Dectin-1*, *Dectin-2*, *DC-SIGN*, *TLRs*, *NOD*, *MelLec*, *MBL*) [7,8,9,10,11,12,13,14], cytokines (*IFNG*, *IL1* gene cluster, *IL6*, *IL8*, *IL10*, *IL12B*) [15,16,17], chemokines (*CXCL10*) [18], and cell surface receptors (*TNFR1*, *TNFR2*, *CX3CR1*) [19,20,21,22,23], whereas only a few studies have addressed the role of intracellular signaling downstream of PRRs [22,24,25], effector T lineage factors, or master regulators of cytokine biosynthesis [26]. 

Several investigations have demonstrated that TNFSF4 (also known as OX40L) expression is upregulated in *Aspergillus fumigatus* sensitized mice [27]. TNFSF4 mediates adhesion of activated T cells to endothelial cells during the infection, which is a key process to induce the expression of leukocyte adhesion molecules (E-selectin, VCAM-1) and secretion of proinflammatory cytokines [28]. In addition, it has been reported that, after activation by thymic stromal lymphopoietin (TSLP), the ligation of TNFSF4 during the interaction of T and dendritic cells (DCs) is a crucial process for clonal expansion of antigen-specific T-cells and generation of memory T cells in response to *A. fumigatus* [29]. On the other hand, it has been reported that MAPKAPK2, a downstream substrate of the p38MAPK (also known as MK2), is a key factor in the modulation of cytokine production during infection by phosphorylating and inactivating the mRNA-destabilizing and translation-inhibiting protein tristetraprolin (TTP) [30]. In addition, it has been reported that *MAPKAPK2* protein is implicated in the modulation of LPS-induced macrophage activation and acute lung injury by regulating let-7e miRNA [31] and likely by inhibiting TTP that controls the posttranscriptional repression of cytokine biosynthesis in macrophages [32]. 

Considering the above-reported findings, we decided to investigate whether 36 single nucleotide polymorphisms (SNPs) within the *TNFSF4* and *MAPKAPK2* loci could influence the risk of developing IA by modulating immune responses and/or immune cell populations. For that purpose, we conducted a two-stage candidate gene association study including 991 hematological patients, of whom 164 were diagnosed with proven or probable IA. In order to shed light into the molecular mechanisms underlying the most interesting associations, we also investigated the role of the *TNFSF4* and *MAPKPAK2* variants in modulating immune responses against *Aspergillus fumigatus* conidia. Furthermore, we evaluated whether *TNFSF4* and *MAPKPAK2* SNPs correlated with a panel of 103 serological and plasmatic inflammatory proteins, 7 serum steroid hormones, and counts of 91 blood-derived immune cell populations. Given the key role of macrophages in the host defense against *A. fumigatus*, we also evaluated whether selected SNPs could correlate with fungicidal activity of macrophages in an independent cohort of 108 healthy blood donors. 

## 2. Methods

### 2.1. Discovery and Replication Populations

A total of 911 hematological patients at high risk of IA were included in this two-stage case control study. All patients were allotransplanted or diagnosed with medullar aplasia, acute myeloid leukemia, or acute lymphoid leukemia receiving intensive chemotherapy regimens. The discovery population consisted of 423 European hematological patients, 67 diagnosed with proven or probable IA and 356 disease-matched patients without any sign of infection and lacking pulmonary infiltrate for a period of, at least, 12 months. Patients diagnosed with possible IA (unreliable diagnosis) were excluded from the study. The replication population included 488 disease-matched patients, 97 of those diagnosed with proven or probable IA. Both discovery and replication populations were ascertained through the aspBIOmics consortium (www.aspbiomics.eu) that aims to identify and to characterize new biomarkers for an effective management of IA using a broad range of approaches including genetic association studies but also RNA, DNA, and proteome analysis of *A. fumigatus* and multiple-ELISA assays and transcriptome analysis of immune cells ex vivo. Demographic and clinical characteristics of the hematological patients included in the discovery and replication cohorts have been reported in detail elsewhere [22,24,33]. The ethical committees of all participating centers and hospitals approved the study: Virgen de las Nieves University hospital (0702/12, Granada, Spain), University Hospital of Salamanca (NCT01742026, Salamanca, Spain), Clinic University Hospital of Valencia (NCT01742026, Valencia, Spain), Centro Nacional de Microbiología (NCT01742026, Madrid, Spain), University of Würzburg (173/11, Würzburg, Germany), Medical University of Innsbruck (UN4529 and 04/2014, Innsbruck, Austria), Università Cattolica del S. Cuore (0029458/16 and 0003932/17, Rome, Italy), and University of Modena and Reggio Emilia (2629/16, Modena, Italy). Approval for the functional genomics studies was obtained from the Arnhem-Nijmegen Ethical Committee (42561.091.12, Nijmegen, The Netherlands) and from the Ethics Subcommittees for Life and Health Sciences of the University of Minho (SECVS, 014/2015, Braga, Portugal), and the National Commission for the Protection of Data (1950/015, Braga, Portugal). In accordance with the Declaration of Helsinki, all participants provided their written informed consent to participate in the study. Proven and probable IA cases were diagnosed by experienced physicians and pathologists according to the 2008 EORTC/MSG criteria [34] and all centers used galactomannan as microbiological criteria for IA diagnosis. 

### 2.2. DNA Extraction, SNP Selection, Genotyping, and Quality Control

Genomic DNA from hematological patients at high risk of IA was extracted from saliva or blood samples using the Oragene^®^-DNA Self-Collection kit (Oragene) or the QIAamp DNA Blood Mini kit (Valencia, CA, EEUU) according to manufacturer’s instructions. Common tagging SNPs (MAF > 5% and r^2^ ≥ 0.8) were selected for the *MAPKAPK2* and *TNFSF4* loci using Haploview according to the Gabriel et al. method [35]. Given the large size of the *TNFSF4* gene and the high number of tagging SNPs found, we decided to select for genotyping only those tagging *TNFSF4* SNPs with a predicted biological function according to the data publicly available in the integrated Regulome database (www.regulomedb.org/), and eQTL browsers (www.gtexportal.org/home/ and https://genenetwork.nl/bloodeqtlbrowser/). A total of 36 SNPs were selected for genotyping in the discovery and replication cohorts (Supplementary Table S1). Genotyping of selected SNPs was performed using KASP^®^ and Taqman^®^ probes according to manufacturer’s instructions (LGC Genomics, Hoddesdon, UK). For quality control, ~5% of DNA samples were randomly included as duplicates and concordance between duplicate samples was ≥99.0%.

### 2.3. Hardy–Weinberg Equilibrium, Genetic Association Analysis, and Meta-Analysis

Deviation from Hardy–Weinberg equilibrium (HWE) was tested in controls (non-IA cases) by chi-square (χ^2^), and logistic regression adjusted for age, sex, and country of origin was used to assess the associations of the *TNFSF4* and *MAPKAPK2* polymorphisms with IA risk assuming log-additive, dominant, and recessive models of inheritance. Those SNPs with the lowest *p*-value in the discovery population according to each genetic model were advanced for replication and meta-analysis of the two populations using a fixed effect model was performed to validate the association observed. Correction for multiple testing was performed using the Bonferroni method but also considering the two inheritance models tested (the log-additive/dominant that showed collinearity and the recessive model). According to this strategy, the significant threshold for the meta-analysis was set to 0.000694 (0.05/36SNPs/2models). Overall statistical power was calculated using Quanto (v.12.4) assuming a log-additive model and a baseline risk of 3% for hematological patients [36]. 

### 2.4. Linkage Disequilibrium (LD) and Haplotype Analysis

Haplotype frequencies were estimated using the expectation–maximization (EM) algorithm and haplotypes were reconstructed using SNPtool and Haploview and block structures were determined according to the method of Gabriel et al. [35]. Haplotype association analysis was performed using the haplo.stats package and association estimates were adjusted for age, sex, and country of origin.

### 2.5. Cell Isolation and Differentiation and Functional Analysis of the TNFSF4 and MAPKAPK2 Variants

With the aim of determining whether those SNPs associated with IA risk had a role in modulating immune responses, we performed in vitro stimulatory experiments and we measured cytokine production (IFNγ, IL1Ra, IL1β, IL6, IL8, IL10, TNFα, IL17, and IL22) after stimulation of peripheral blood mononuclear cells (PBMCs), whole blood, or monocyte-derived macrophages (MDM) from 408 healthy subjects of the 500FG from the Human Functional Genomics Project (HFGP) with *Aspergillus fumigatus* conidia. Given the impact of steroid hormones in modulating immune responses, we also evaluated the correlation of SNPs with serum levels of 7 steroid hormones (androstenedione, cortisol, 11-deoxy-cortisol, 17-hydroxy progesterone, progesterone, testosterone, and 25 hydroxy vitamin D3) in a subset of the HFGP subjects without hormonal replacement therapy or oral contraceptives (*n* = 280). After log transformation, correlation between SNPs and cytokine expression quantitative trait loci (cQTLs) or serum steroid hormone levels was evaluated using linear regression adjusted for age and sex in R (http://www.r-project.org/). Significance thresholds were set to 0.00185 and 0.0024 (0.05/3SNPs/9cytokines-7hormones) for cQTL and steroid hormone analysis, respectively.

### 2.6. Correlation between TNFSF4 and MAPKAPK2 Polymorphisms and Cell Counts of 91 Blood-Derived Immune Cell Populations and Serum/Plasmatic Proteomic Profile

We also investigated whether *TNFSF4* and *MAPKAPK2* polymorphisms had an impact on blood cell counts by analyzing a set of 91 manually annotated immune cell populations and genotype data from the HFGP cohort that included 408 healthy subjects (Supplementary Table S2). Cell populations were measured by 10-color flow cytometry (Navios flow cytometer, Beckman Coulter, Miami, Florida, USA) after blood sampling (2–3 h) and cell count analysis was performed using the Kaluza software (Beckman Coulter, v.1.3). In order to reduce inter-experimental noise and increase statistical power cell count, analysis was performed by calculating parental and grandparental percentages, which were defined as the percentage of a certain cell type within the subpopulation of cells from which it was isolated [37]. Detailed laboratory protocols for cell isolation, reagents, gating, and flow cytometry analysis have been reported elsewhere [38] and the accession number for the raw flow cytometry data and analyzed data files are available upon request to the authors (http://hfgp.bbmri.nl). A proteomic analysis was also performed in serum and plasma samples from the HFGP cohort. Circulating proteins were measured using the commercial Olink^®^ Inflammation panel (Olink, Uppsala, Sweden) that resulted in the measurement of 103 different biomarkers (Supplementary Table S3). Protein levels were expressed on a log2-scale as normalized protein expression values, and normalized using bridging samples to correct for batch variation. Correlation between SNPs and protein levels or blood cell counts was evaluated using linear regression adjusted for age and sex in R. Considering the number of proteins (*n* = 103), cell populations (*n* = 91), and polymorphisms (*n* = 3) tested, *p*-values of 0.00016 and 0.000183 were set as significant thresholds for the proteomic and cell-level variation analysis.

### 2.7. Assessment of Fungicidal Activity

Finally, we also analyzed the impact of selected SNPs on fungicidal activity in human monocyte-derived macrophages (MDM) from an independent cohort including 108 healthy subjects. For that purpose, human MDM were infected with live *A. fumigatus* conidia at an effector-to-target ratio of 1:10 for 1 h. After removing the non-ingested conidia, MDM were allowed to kill internalized conidia for 2 h. To measure their fungicidal ability, MDM were lysed by quickly freezing at −80 °C and thawing at 37 °C (releasing ingested conidia) and cell lysates were mixed thoroughly, and serial dilutions were made in phosphate buffered solution (PBS) and plated on Sabouraud dextrose agar. Following a 2-day incubation, the number of colony-forming units (CFU) was enumerated and the percentage of CFU inhibition was calculated. CFU counts of conidia treated in the same experimental conditions but in the absence of macrophages were used as controls. Detailed information about fungicidal experiments has been previously reported in detail [22,24,33]. Statistical significance of in vitro fungicidal experiments was evaluated using an unpaired t-test with or without Welch’s correction or Mann–Whitney U test. A *p* ≤ 0.05 was considered significant (Prism v6.0). 

### 2.8. In Silico Functional Analysis

Besides the functional analysis of the *TNFSF4* and *MAPKAPK2* polymorphisms reported above, we also used the HaploReg SNP annotation tool to further investigate the functional consequences of each specific variant (http://www.broadinstitute.org/mammals/haploreg/haploreg.php). Finally, we assessed whether any of the potentially interesting markers correlated with mRNA expression levels of their respective genes using data from the GTex portal (www.gtexportal.org/home/).

## 3. Results

### 3.1. Characteristics of Study Subjects

Overall, this two-stage case control study included 911 hematological patients at high risk of IA. Of those, a total of 164 patients were diagnosed with proven or probable IA. Demographic and clinical characteristics of the hematological patients included are summarized in Table 1. Briefly, IA and non-IA groups had a similar age and underlying disease distribution in both the discovery and replication cohorts. In addition, both IA and non-IA groups showed a similar proportion of patients who underwent allogeneic stem cell transplantation in both study cohorts, as shown in Table 1. Nonetheless, IA was more frequently found in men than in women (*p* = 0.001) and, as expected, the use of antifungal prophylaxis was less frequent among those patients diagnosed with IA (53.85% vs. 66.79%, *p* = 0.008; Table 1). 

### 3.2. Association Analysis and Functional Impact of TNFSF4 and MAPKAPK2 Polymorphisms

All SNPs analyzed were in HWE (*p* < 0.001) in the control group (non-IA cases). In the discovery population, logistic regression analyses adjusted for age, sex, and country of origin revealed that each copy of the *TNFSF4*_rs4357565C_, *TNFSF4*_rs7526628T_, *MAPKAPK2*_rs12126682G_, *MAPKAPK2*_rs11119267C_, *MAPKAPK2*_rs12123706T_, and *MAPKAPK2*_rs12137965G_ alleles increased the risk of developing IA by 0.5 to 2-fold (OR = 1.47–2.06), whereas each copy of the *MAPKAPK2*_rs17013271T_ and *MAPKAPK2*_rs4845123A_ alleles decreased the risk of infection (OR = 0.48 and 0.59; Table 2). 

Interestingly, we observed that most of these SNPs also impacted on IA risk according to other models of inheritance (dominant or recessive), which reinforced the idea of a true effect of the *TNFSF4* and *MAPKAPK2* loci in modulating disease risk. Considering these potentially interesting findings, we decided to advance for replication those *TNFSF4* and *MAPKAPK2* SNPs that showed significant results in the discovery population. Importantly, the meta-analysis of the discovery and replication cohorts confirmed a consistent association of the *TNFSF4*_rs7526628T/T_ genotype with an increased risk of developing IA that remained statistically significant after correction for multiple testing (OR_Meta-recessive_ = 2.25, 95% CI 1.46–3.46, *p* = 0.00022; Table 3). Even though it did not survive multiple testing correction, we also found that carriers of the *TNFSF4*_rs7526628T_ allele had an increased risk of developing the infection, which indicated that a single copy of the risk allele might be sufficient to drive disease susceptibility (OR_Meta-dominant_ = 1.52, 95% CI 1.17–1.96, *p* = 0.0015; Table 3).

**Figure 1:**
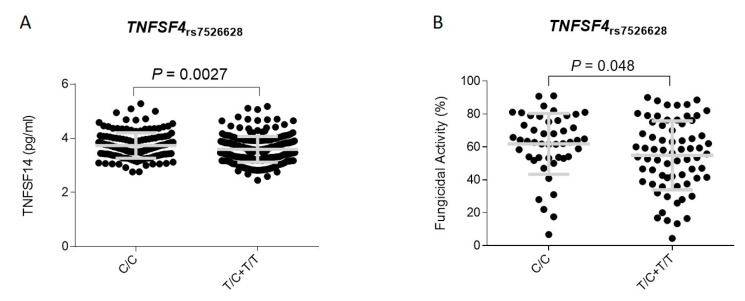
Correlation between the *TNFSF4*_rs7526628_ SNP and serum levels of TNFSF14 (pg/mL) (**A**) and fungicidal activity (%) of monocyte-derived macrophages (**B**). Grey line represents the mean with error bars (standard deviation).

In support of the hypothesis suggesting a role of the *TNFSF4* in modulating IA risk, the proteomic serum analysis of the 500FG cohort from the HFGP showed that carriers of the *TNFSF4*_rs7526628T_ allele had decreased serum concentration of TNFSF14 protein (*p* = 0.0027; Figure 1A), a membrane-bound protein transiently expressed on activated T cells, natural killer (NK) cells, and immature dendritic cells that is involved in promoting proinflammatory responses in multiple contexts and induces T cell proliferation and lung tissue remodeling. In line with these findings, we also found in an independent cohort of healthy donors that macrophages from carriers of the *TNFSF4*_rs7526628T_ allele showed a decreased fungicidal activity against *A. fumigatus* conidia when compared with those harboring the most common *TNFSF4*_rs7526628C/C_ genotype (*P*_Dominant_ = 0.048; Figure 1B). Although we did not find any positive correlation between this SNP and cytokine production (IFNγ, IL1Ra, IL1β, IL6, IL8, IL10, TNFα, IL17, and IL22) after stimulation of peripheral blood mononuclear cells (PBMCs) with *A. fumigatus* conidia, these results together with our genetic findings pointed to a weak but still functional effect of the *TNFSF4*_rs7526628_ SNP that might influence the killing activity of macrophages and TNFSF14-mediated T cell proliferation and lung tissue remodeling. 

**Table 2:** Association analysis of selected SNPs within the *TNFSF4* and *MAPKAPK2* loci and IA risk in the discovery cohort.

SNP_rsID	Gene	Risk Allele	OR (95% CI) ^δ^	*p*	OR (95% CI) ^Ϯ^	*p*	OR (95% CI) ^¥^	*p*
rs10489269	TNFSF4	A	1.23 (0.56–2.70)	0.61	1.08 (0.47–2.51)	0.86	NA (NA-NA)	NA
rs7518045	TNFSF4	G	1.08 (0.54–2.15)	0.83	0.91 (0.41–1.98)	0.80	NA (NA-NA)	NA
rs10489266	TNFSF4	G	0.56 (0.27–1.17)	0.10	0.58 (0.27–1.26)	0.15	NA (NA-NA)	NA
rs2205959	TNFSF4	G	0.85 (0.57–1.27)	0.44	1.11 (0.61–2.00)	0.73	0.46 (0.20–1.09)	0.058
rs61828280	TNFSF4	G	0.63 (0.30–1.32)	0.20	0.66 (0.30–1.42)	0.27	NA (NA-NA)	NA
rs4916320	TNFSF4	G	0.76 (0.40–1.44)	0.38	0.77 (0.40–1.49)	0.43	NA (NA-NA)	NA
rs4357565	TNFSF4	C	**1.73 (1.13–2.63)**	**0.011**	**2.13 (1.21–3.74)**	**0.0077**	1.69 (0.67–4.24)	0.28
rs1342038	TNFSF4	A	0.68 (0.44–1.03)	0.065	0.73 (0.41–1.30)	0.29	0.40 (0.16–1.02)	0.056
rs947505	TNFSF4	A	1.17 (0.68–2.01)	0.58	1.07 (0.60–1.92)	0.82	4.17 (0.62–28.3)	0.17
rs56307807	TNFSF4	C	0.95 (0.45–2.02)	0.90	0.85 (0.38–1.94)	0.70	4.32 (0.26–72.4)	0.33
rs6425219	TNFSF4	T	1.08 (0.72–1.63)	0.71	1.07 (0.62–1.87)	0.80	1.19 (0.51–2.76)	0.69
rs1418191	TNFSF4	C	1.47 (0.85–2.54)	0.18	1.63 (0.88–3.00)	0.13	0.87 (0.09–8.34)	0.90
rs1578624	TNFSF4	C	0.86 (0.58–1.27)	0.44	0.73 (0.41–1.29)	0.29	0.97 (0.49–1.92)	0.92
rs7526628	TNFSF4	T	**1.72 (1.16–2.55)**	**0.0072**	**1.87 (1.02–3.42)**	**0.038**	**2.27 (1.22–4.20)**	**0.0068**
rs7549074	TNFSF4	C	1.43 (0.90–2.26)	0.14	1.34 (0.77–2.31)	0.30	2.95 (0.90–9.66)	0.089
rs6700269	TNFSF4	T	1.74 (0.97–3.12)	0.075	1.94 (0.9–3.77)	0.059	1.56 (0.17–14.2)	0.71
rs12140760	TNFSF4	A	0.83 (0.56–1.21)	0.33	0.79 (0.45–1.37)	0.40	0.75 (0.35–1.58)	0.44
rs17013271	DYRK3	T	**0.48 (0.32–0.73)**	**0.0004**	**0.48 (0.28–0.83)**	**0.0087**	**0.18 (0.05–0.59)**	**0.0005**
rs6540512	DYRK3	A	1.29 (078–2.11)	0.32	1.47 (0.83–2.58)	0.19	0.52 (0.06–4.37)	0.51
rs12126682	DYRK3	G	**1.47 (1.01–2.14)**	**0.044**	1.62 (0.89–2.97)	0.11	1.77 (0.94–3.34)	0.084
rs4845123	DYRK3	A	**0.59 (0.38–0.90)**	**0.012**	0.60 (0.35–1.03)	0.063	**0.27 (0.08–0.89)**	**0.012**
rs17435120	DYRK3|MAPKAPK2	G	1.42 (0.98–2.06)	0.065	1.44 (0.80–2.58)	0.21	1.85 (0.98–3.49)	0.065
rs12407425	DYRK3|MAPKAPK2	A	0.85 (0.46–1.58)	0.61	0.81 (0.41–1.59)	0.53	1.29 (0.14–11.8)	0.82
rs11119267	DYRK3|MAPKAPK2	C	**1.49 (1.00–2.22)**	**0.047**	**2.40 (1.26–4.59)**	**0.0051**	1.07 (0.51–2.25)	0.86
rs12038489	DYRK3|MAPKAPK2	A	1.03 (0.58–1.81)	0.92	1.08 (0.57–2.04)	0.82	0.67 (0.07–6.02)	0.71
rs61815610	MAPKAPK2	T	0.79 (0.41–1.52)	0.47	0.82 (0.42–1.62)	0.56	NA (NA-NA)	NA
rs10863788	MAPKAPK2	A	0.73 (0.49–1.09)	0.12	0.83 (0.48–1.44)	0.51	**0.37 (0.14–0.98)**	**0.027**
rs12123706	MAPKAPK2	T	**1.74 (1.12–2.70)**	**0.014**	**2.35 (1.35–4.10)**	**0.0022**	0.86 (0.24–3.12)	0.81
rs4072677	MAPKAPK2	G	0.85 (0.58–1.24)	0.39	0.92 (0.52–1.63)	0.77	0.65 (0.31–1.35)	0.23
rs61815626	MAPKAPK2	A	**0.35 (0.10–1.17)**	**0.049**	0.34 (0.10–1.18)	0.054	NA (NA-NA)	NA
rs12030124	MAPKAPK2	T	1.21 (0.72–2.03)	0.48	1.29 (0.72–2.28)	0.40	0. (0.09–6.73)	0.81
rs4256810	MAPKAPK2	C	0.72 (0.46–1.13)	0.14	0.75 (0.44–1.29)	0.29	0.36 (0.08–1.56)	0.76
rs28394820	MAPKAPK2	C	0.80 (0.47–1.35)	0.39	0.88 (0.50–1.55)	0.65	NA (NA-NA)	NA
rs4073250	MAPKAPK2	T	0.69 (0.43–1.11)	0.12	0.70 (0.39–1.25)	0.22	0.35 (0.08–1.55)	0.11
rs7515374	MAPKAPK2||IL10	C	0.86 (0.58–1.28)	0.46	0.88 (0.50–1.54)	0.66	0.71 (0.58–1.28)	0.46
rs12137965	MAPKAPK2||IL10	G	**2.06 (1.34–3.16)**	**0.0011**	**3.01 (1.68–5.38)**	**0.0001**	1.38 (0.48–3.95)	0.56

All analyses were adjusted for age, sex, and country of origin. *p* < 0.05 in bold. **^δ^** Logistic regression analysis assuming a log-additive model of inheritance. **^Ϯ^** Logistic regression analysis assuming a dominant model of inheritance. **^¥^** Logistic regression analysis assuming a recessive model of inheritance.

**Table 3:** Association analysis of the most significant SNPs within *TNFSF4* and *MAPKAPK2* regions in the meta-analysis of study cohorts.

		Discovery Population (*n* = 423)		Replication Population (*n* = 488)		Meta-Analysis (*n* = 911)		
SNP_rsID	Risk Allele	OR (95% CI) ^δ^	*p*	OR (95% CI) ^δ^	*p*	OR (95% CI) ^δ^	*p*	*P_Het_*
rs4357565	C	**1.73 (1.13–2.63)**	**0.011**	0.95 (0.64–1.42)	0.81	1.26 (0.94–1.68)	0.118	**0.043**
rs4357565	C	1.69 (0.67–4.24) ^¥^	0.28	1.31 (0.55–3.11) ^¥^	0.55	1.48 (0.79–2.78) ^¥^	0.227	0.693
rs7526628	T	**1.72 (1.16–2.55)**	**0.0072**	1.38 (0.98–1.94)	0.068	**1.52 (1.17–1.96)**	**0.0015**	0.41
rs7526628	T	**2.27 (1.22–4.20) ^¥^**	**0.0068**	**2.23 (1.22–4.06) ^¥^**	**0.011**	**2.25 (1.46–3.46) ^¥^**	**0.00022**	0.97
rs17013271	T	**0.48 (0.32–0.73)**	**0.0004**	0.85 (0.58–1.23)	0.38	**0.66 (0.50–0.87)**	**0.0029**	0.05
rs17013271	T	**0.48 (0.28–0.83) ^Ϯ^**	**0.0087**	0.73 (0.44–1.23) ^Ϯ^	0.24	**0.60 (0.41–0.87) ^Ϯ^**	**0.0071**	0.27
rs12126682	G	**1.47 (1.01–2.14)**	**0.044**	0.82 (0.56–1.19)	0.29	1.10 (0.84–1.43)	0.486	**0.032**
rs12126682	G	1.62 (0.89–2.97) ^Ϯ^	0.11	0.81 (0.48–1.34) ^Ϯ^	0.41	1.08 (0.73–1.60)	0.685	0.086
rs11119267	C	**1.49 (1.00–2.22)**	**0.047**	1.40 (0.97–2.02)	0.069	**1.44 (1.10–1.89)**	**0.0080**	0.822
rs11119267	C	**2.40 (1.26–4.59) ^Ϯ^**	**0.0051**	1.55 (0.86–2.78) ^Ϯ^	0.13	**1.89 (1.22–2.92)**	**0.0040**	0.326
rs12123706	T	**1.74 (1.12–2.70)**	**0.014**	0.84 (0.55–1.29)	0.43	1.20 (0.88–1.62)	0.254	**0.020**
rs12123706	T	**2.35 (1.35–4.10) ^Ϯ^**	**0.0022**	0.88 (0.52–1.48) ^Ϯ^	0.63	1.40 (0.95–2.04)	0.086	**0.012**
rs12137965	G	**2.06 (1.34–3.16)**	**0.0011**	1.28 (0.85–1.91)	0.24	**1.60 (1.19–2.15)**	**0.0017**	0.11
rs12137965	G	**3.01 (1.68–5.38) ^Ϯ^**	**0.0001**	1.26 (0.77–2.08) ^Ϯ^	0.36	**1.81 (1.25–2.65) ^Ϯ^**	**0.0019**	**0.030**

**Figure 2:**
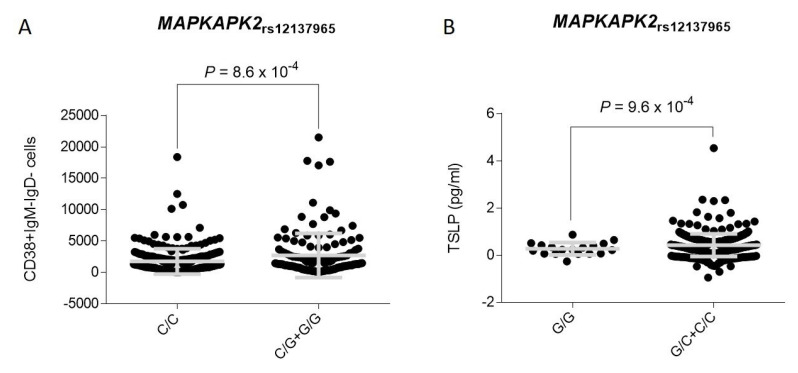
Correlation between the *MAPKAPK2*_rs12137965_ SNP and blood CD38+IgM-IgD- B cells (**A**) and serum levels of thymic stromal lymphopoietin (TSLP) (pg/mL) (**B**). Grey line represents the mean with error bars (standard deviation). Likewise, we observed that carriers of the protective *MAPKAPK2*_rs17013271T_ allele had decreased numbers of CD27-IgM-IgD- B cells (*p* = 0.00087; Figure 3A) but also multiple B cell subpopulations including CD27-IgM+, CD19+CD3-, CD19+CD20-, CD38+CD24+, and naïve IgM+IgD+ B cells (Supplementary Table S4). Intriguingly, we found that carriers of the *MAPKAPK2*_rs17013271T_ allele showed significantly decreased numbers of CD14+ cells (*p* = 0.00018; Figure 3B) and classical monocytes (defined as CD14+CD16- cells; *p* = 0.00023; Figure 3C) in blood. Although only the association of the *MAPKAPK2*_rs17013271T_ allele with decreased numbers of CD14+ cells remained statistically significant after multiple testing correction (*p* ≤ 0.00018), altogether these results suggested a plausible role of the *MAPKAPK2* locus in determining IA risk likely though the modulation of antigen presenting cell counts in blood (including monocytes and B cells) and the inhibition of TSLP-mediated Th2 immune responses but also the promotion of humoral immunity.

All analyses were adjusted for age, sex, and country of origin. *p* < 0.05 in bold. **^δ^** Logistic regression analysis assuming a log-additive model of inheritance. **^Ϯ^** Logistic regression analysis assuming a dominant model of inheritance. **^¥^** Logistic regression analysis assuming a recessive model of inheritance.On the other hand, the meta-analysis of the discovery and replication study cohorts showed that each copy of the *MAPKAPK2*_rs12137965G_ allele increased the risk of developing IA by 60% (OR_Meta-additive_ = 1.60, 95% CI 1.19–2.15, *p* = 0.0017). In line with this finding, we found that carriers of the *MAPKAPK2*_rs11119267C_ allele had an increased risk of developing the infection (OR_Meta-dominant_ = 1.89, 95% CI 1.22–2.92, *p* = 0.0040; Table 3) whereas each copy of the *MAPKAPK2*_rs17013271T_ allele was estimated to decrease the risk of developing IA (OR_Meta-additive_ = 0.66, 95% CI 0.50–0.87, *p* = 0.0029; Table 3). The effect of the *MAPKAPK2*_rs17013271_ SNP on IA risk was also observed when a dominant model of inheritance was assumed (C/C vs. C/T+T/T), which reinforced the role of the *MAPKAPK2* gene in modulating IA risk (OR_Meta-dominant_ = 0.60, 95% CI 0.41–0.87, *p* = 0.0071; Table 3). Although none of these genetic associations remained significant after correction for multiple testing, we found that carriers of the risk *MAPKAPK2*_rs12137965G_ allele showed increased levels of CD38+IgM-IgD- plasmablasts in blood (*p* = 0.00086; Figure 2A). In addition, we observed that carriers of the *MAPKAPK2*_rs12137965G/G_ genotype also showed decreased levels of thymic stromal lymphopoietin (TSLP) in serum (*p* = 0.00097; Figure 2B).

## 4. Discussion

This study reports for the first time the association of *TNFSF4* and *MAPKAPK2* polymorphisms with the risk of developing IA infection. The most significant association with IA risk was found for the *TNFSF4*_rs7526628_ polymorphism in the second intron of the *TNFSF4* locus. Thus, carriers of the *TNFSF4*_rs7526628T/T_ genotype had a 2-fold increased risk of IA when compared with those harboring the most common allele. *TNFSF4* gene maps on chromosome 1q25 and encodes for a costimulatory molecule expressed mostly in mature DCs, B cells, and macrophages [39] that is essential to acquire antigen presenting cell function [40]. Besides antigen presenting cells, *TNFSF4* expression has also been found in regulatory T (Treg) cells where it promotes cell development and regulates cell homeostasis, and Treg suppressive activity thus controlling T-cell tolerance [41]. In addition, TNFSF4 has been implicated in the control of Th2 priming, effector cell function, cell survival, and memory cell induction that drive lung inflammation [42,43]. Previous studies have also suggested that *TNFSF4* expression is upregulated during infection [44] and that the presence of genetic polymorphisms within the *TNFSF4* locus is associated with the risk of developing immune-related diseases [45,46] and determining stem cell transplantation outcomes [47]. Given that only the *TNFSF4*_rs7526628_ SNP showed a consistent effect on IA risk in both the discovery and replication populations, we hypothesize that this SNP might be the causative variant influencing innate immune responses against *A. fumigatus*. In support of this notion, we found that macrophages from carriers of the *TNFSF4*_rs7526628T_ allele showed a decreased fungicidal activity when compared with those carrying the most common genotype. In addition, we found that carriers of the *TNFSF4*_rs7526628T/T_ genotype showed decreased serum levels of TNFSF14, a protein that has been shown to be involved in the development of pro-inflammatory responses but also in lung tissue remodeling [48] and lung fibrosis [49]. A growing body of evidence suggests that TNFSF14 through interaction with its receptors, including herpes virus entry mediator (HVEM) and lymphotoxin beta receptor (LTβR) can induce T cell proliferation, airway remodeling, and proinflammatory responses in a broad range of contexts and, therefore, it is plausible to think that decreased serum levels of this circulating protein might increase susceptibility to IA. In line with the hypothesis suggesting a role of the TNFSF4 locus in determining the response to *A. fumigatus*, Nguyen and coworkers demonstrated that vitamin D3 deficiency, which increased the expression of TNFSF4 on lung CD11C+ cells, was essential for the promotion of Th2 immune responses during *A. fumigatus* infection [50]. Similarly, Kreindler and coworkers reported that vitamin D3 attenuated in a TNFSF4-dependent manner Th2 immune responses to *A. fumigatus* mounted by CD4+T cells from cystic fibrosis patients with allergic bronchopulmonary aspergillosis [51]. Although at this point it was tempting to speculate that the *TNFSF4*_rs7526628_ SNP might be biologically functional and drive susceptibility to IA by inhibiting TNFSF4- and TNFSF14-mediated proinflammatory responses and fungal clearance mediated by macrophages and DCs, we recognize that none of the functional findings attributed to the *TNFSF4*_rs7526628_ SNP remained statistically significant after correction for multiple testing and that, therefore, additional functional studies are still warranted to unravel the exact mechanisms by which this SNP is affecting immune responses against *A. fumigatus*.

Another interesting result of this study was the consistent association of three tagging variants within the *MAPKAPK2|DYRK3* region with the risk of developing IA. The *MAPKAPK2* gene is located downstream of IL10 receptor and within the IL10 gene cluster including the IL10 locus but also those genes encoding for relevant cytokines, such as IL19, IL20, and IL24. The strongest effect on IA risk was found for the *MAPKAPK2*_rs17013271_ and *MAPKAPK2*_rs12137965_ SNPs that are located upstream and downstream of the *MAPKAPK2* gene, respectively. While carriers of the *MAPKAPK2*_rs12137965G_ allele had an increased risk of developing IA, those harboring the *MAPKAPK2*_rs17013271T_ allele showed a decreased risk of developing the infection. Mechanistically, we found that carriers of the risk *MAPKAPK2*_rs12137965G_ allele showed an increased number of CD38+IgM-IgD- B cells, whereas carriers of the *MAPKAPK2*_rs12137965G/G_ genotype showed decreased serum levels of TSLP, a protein expressed by human airway epithelial cells [52] and smooth muscle cells [53] but also DCs [54]. TSLP expression has been associated with the activation of NF-κB downstream of toll-like receptors (TLRs) [52,55], NOD2 expression [56], and stimulation of human airway smooth muscle cells with IL1β and TNFα [53], as well as pulmonary infections [57,58]. Furthermore, TSLP has been implicated in multiple immune processes including maturation of blood CD11C+ dendritic cells, induction of TNFSF4 protein expression on DCs to prime naive CD4+ T cells to differentiate into proinflammatory Th2 cells [59], maintenance and expansion of Th2 central memory T cells [60], and differentiation of Treg cells [61] but also B lymphopoiesis [62]. In addition, TSLP expression is dependent on NK-κB and AP-1 [63] but also the p38MAPK/MAPKAPK2 signaling pathway [53] in such a way that suppression of the p38MAPK drastically decreases TSLP secretion after the stimulation with IL1β and TNFα [53]. Given that overexpression of TSLP inhibits B lymphopoiesis [62], induces *TNFSF4*-mediated Th2 immune responses, and given that its production is controlled by the p38MAPK/MAPKAPK2 pathway, it seems conceivable to suggest that the presence of SNPs within the *MAPKAPK2* locus that correlate with serum TSLP and blood B cell levels might influence the risk of developing IA. In support of this hypothesis, we also found that carriers of the *MAPKAPK2*_rs17013271T_ allele, who had a decreased risk of developing IA, showed lower blood numbers of CD27-IgM-IgD- B cells (among other B cell subpopulations) when compared with those carrying the most common genotype, but also significantly lower numbers of CD14+ and CD14+CD16- monocytes, which reinforced the idea of a role of the *MAPKAPK2* gene in modulating myeloid cell proliferation. In line with our findings, an interesting study demonstrated that the activation of both p38MAPK and MAPKAPK2 was required for B cell proliferation and that the inhibition of p38MAPK activity in vivo with a cell-permeable inhibitor (SB203580), under conditions that prevented MAPKAPK2 activation, strongly perturbed CD40-induced B cell proliferation and TNFα-induced NFκB activation [64]. Similarly, Sutherland and coworkers (1996) reported that upon cross-linking of CD40, MAPKAPK2 was able to promote B cell survival [65]. In addition, although the role of *MAPKAPK2* in the modulating B cell proliferation during infection is poorly studied, the use of SB203580 was shown to reduce *Dengue virus-*induced liver inflammation and tissue injury by decreasing *MAPKAPK2*-dependent cytokine (TNFα, IL6, and IL10) and chemokine (RANTES and IP-10) production [66]. In addition, another study showed that miR-125a, which is involved in determining the commitment and immunosuppressive capacity of Treg cells, was highly expressed in a model of parasitic infection and that it mediated its action through the regulation of both *TNFSF4* and *MAPKAPK2* genes [67]. Considering our genetic and functional results but also those reported in the literature, we suggest that the presence of functional polymorphisms within the *TNFSF4* and *MAPKAPK2* genes influence the risk of developing IA in susceptible hosts by modulating TNFSF14- and TSLP-mediated immune responses but also myeloid cell proliferation. However, although we have provided some insights about the functional role of the *TNFSF4* and *MAPKAPK2* polymorphisms in determining the risk of IA, we believe that additional studies are still warranted to identify the exact mechanisms by which these loci modulate the risk of fungal infection. In silico data from HaploReg showed that the *TNFSF4*_rs7526628_, *MAPKAPK2*_rs12137965_, and *MAPKAPK2*_rs17013271_ SNPs were located among histone marks in multiple immune cell types including primary T CD8+ naïve cells, T helper, and T regulatory cells but also primary B cells. In addition, in silico analysis showed that the *MAPKAPK2*_rs12137965_ and *MAPKAPK2*_rs17013271_ SNPs were involved in determining chromatin states on these cells, which reinforced the idea of a functional effect of these SNPs to modulate the risk of IA infection. 

To conclude, it is necessary to mention that this study has both strengths and weaknesses. A strength is the large population size of the aspBIOmics population that allowed a sufficiently powered association analysis to detect small effects. Based on the genotype frequencies observed in the global cohort, we had 80% of power (log-additive model) to detect an OR of 1.76 at alpha = 0.00068 (multiple testing threshold) for an SNP with a minor allele frequency of 0.25. Another strength was the use of a replication cohort that confirmed the same direction of the association of the *TNFSF4* and *MAPKAPK2* SNPs with IA risk in both the discovery and replication populations. In addition, this study included a comprehensive functional analysis conducted in the HFGP cohort that consisted of cQTL analysis after stimulation of whole blood, PBMCs, and MDMs with *A. fumigatus* conidia but also a comprehensive analysis of serological and plasmatic inflammatory biomarkers, serum steroid hormones, and blood-derived immune cells. Functional experiments also included assessment of fungicidal activity on human MDM infected with *A. fumigatus*, which allowed us to directly measure the impact of *TNFSF4* and *MAPKAPK2* SNPs on the efficiency of *A. fumigatus* clearance. Finally, a drawback was the multicenter nature of this study that placed inevitable limitations in clinical data collection that made impossible, for instance, the adjustment of the genetic association analysis for antifungal prophylaxis. 

## Figures and Tables

**Figure 3:**
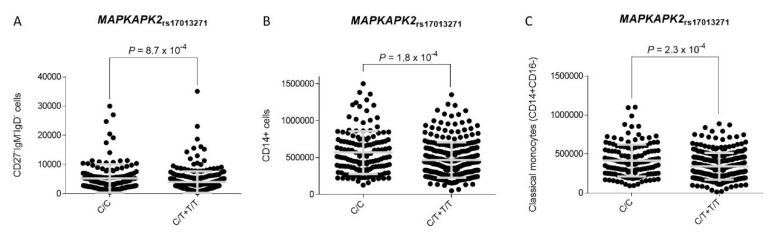
Correlation between the *MAPKAPK2*_rs17013271_ SNP and blood CD27-IgM-IgD- B cells (**A**), blood CD14+ cells (**B**) and classical CD14+CD16- monocytes (**C**).

**Table 1:** Baseline and clinical characteristic of patients with or without invasive aspergillosis (IA).

	Discovery Population			
	Overall (*n* = 423)	IA patients (*n* = 67)	non-IA patients (*n* = 356)	*p* value
Demographic variables				
Age (Average ± SD)	52.35 ± 15.37	53.00 ± 12.69	52.23 ± 15.82	0.663
Sex ratio (male/female)	1.11 (222/200)	1.79 (43/24)	1.02 (179/176)	**0.037**
Hematological disease				
AML	328 (77.54)	49 (73.13)	279 (78.37)	0.346
ALL	58 (13.71)	14 (20.90)	44 (12.36)	0.062
other	37 (08.75)	4 (05.97)	33 (09.27)	0.380
allo-SCT	137 (32.39)	20 (29.85)	117 (32.87)	0.629
Ever received prophylaxis ^†^	244 (64.89)	22 (43.13)	222 (68.31)	**0.0004**
	**Replication population**			
	Overall (*n* = 488)	IA patients (*n* = 97)	non-IA patients (*n* = 391)	*p* value
Demographic variables				
Age (Average ± SD)	50.90 ± 17.12	51.42 ± 15.72	50.76 ± 17.44	0.734
Sex ratio (male/female)	1.32 (278/210)	2.13 (66/31)	1.18 (212/179)	**0.014**
Hematological disease				
AML	317 (64.96)	63 (65.95)	254 (64.96)	0.998
ALL	55 (11.27)	10 (10.31)	45 (11.51)	0.738
other	116 (23.77)	24 (24.74)	92 (23.53)	0.802
allo-SCT	251 (51.43)	54 (55.67)	197 (50.38)	0.351
Ever received prophylaxis ^†^	193 (64.12)	41 (62.12)	152 (64.68)	0.702
	**Overall population**			
	Overall (*n* = 911)	IA patients (*n* = 164)	non-IA patients (*n* = 747)	*p* value
Demographic variables				
Age (Average ± SD)	51.57 ± 16.35	52.07 ± 14.58	51.46 ± 16.71	0.637
Sex ratio (male/female)	1.22 (500/410)	1.98 (109/55)	1.10 (391/355)	**0.001**
Hematological disease				
AML	645 (70.80)	112 (68.29)	533 (71.35)	0.435
ALL	113 (12.40)	24 (14.63)	89 (11.91)	0.339
other	153 (16.80)	28 (17.07)	125 (16.74)	0.916
allo-SCT	388 (42.59)	74 (45.12)	314 (42.03)	0.469
Ever received prophylaxis ^†^	437 (64.55)	63 (53.85)	374 (66.79)	**0.008**

Abbreviations: allo-SCT: Allogeneic stem cell transplantation, AML: acute myeloid leukemia, ALL: acute lymphoid leukemia. *p* ≤ 0.05 was considered significant and shown in bold. **^†^** Prophylaxis status was available in 677 patients, 376 subjects from the discovery cohort (51 IA and 325 non-IA patients) and 301 subjects from the replication cohort (66 IA and 235 non-IA patients). Percentage was calculated according to the number of subjects having prophylaxis information.

## Data Availability

All data used in this project have been meticulously cataloged and archived in the BBMRI-NL data infrastructure (https://hfgp.bbmri.nl/) using the MOLGENIS open-source platform for scientific data [68]. This allows flexible data querying and download, including sufficiently rich metadata and interfaces for machine processing (R statistics, REST API) and using FAIR principles to optimize Findability, Accessibility, Interoperability and Reusability [69]. Genetic data from the aspBIOmics population can be accessed at ftp.genyo.es upon request.

## Supplementary Materials

The following are available online at https://www.mdpi.com/2309-608X/7/1/4/s1, Table S1: Selected SNPs within the *TNFSF4* and *MAPKAPK2* loci, Table S2: Cell types analysed either in whole blood or peripheral mononuclear blood cells, Table S3: Serum and plasma metabolites measured in the HFGP cohort, Table S4: Correlation of *MAPKAPK2* SNPs with blood cell counts according to a dominant model of inheritance.

## References

[1] Baddley J.W., Andes D.R., Marr K.A., Kontoyiannis D.P., Alexander B.D., Kauffman C.A., Oster R.A., Anaissie E.J., Walsh T.J., Schuster M.G. (2010). Factors associated with mortality in transplant patients with invasive aspergillosis. Clin. Infect. Dis..

[2] Steinbach W.J., Marr K.A., Anaissie E.J., Azie N., Quan S.P., Meier-Kriesche H.U., Apewokin S., Horn D.L. (2012). Clinical epidemiology of 960 patients with invasive aspergillosis from the PATH Alliance registry. J. Infect..

[3] Dutkiewicz R., Hage C.A. (2010). Aspergillus infections in the critically ill. Proc. Am. Thorac. Soc..

[4] Maertens J.A., Raad I.I., Marr K.A., Patterson T.F., Kontoyiannis D.P., Cornely O.A., Bow E.J., Rahav G., Neofytos D., Aoun M. (2016). Isavuconazole versus voriconazole for primary treatment of invasive mould disease caused by Aspergillus and other filamentous fungi (SECURE): A phase 3, randomised-controlled, non-inferiority trial. Lancet.

[5] Lionakis M.S., Levitz S.M. (2018). Host Control of Fungal Infections: Lessons from Basic Studies and Human Cohorts. Annu. Rev. Immunol..

[6] Cunha C., Aversa F., Romani L., Carvalho A. (2013). Human genetic susceptibility to invasive aspergillosis. PLoS Pathog..

[7] Cunha C., Aversa F., Lacerda J.F., Busca A., Kurzai O., Grube M., Loffler J., Maertens J.A., Bell A.S., Inforzato A. (2014). Genetic PTX3 deficiency and aspergillosis in stem-cell transplantation. N. Engl. J. Med..

[8] Fisher C.E., Hohl T.M., Fan W., Storer B.E., Levine D.M., Zhao L.P., Martin P.J., Warren E.H., Boeckh M., Hansen J.A. (2017). Validation of single nucleotide polymorphisms in invasive aspergillosis following hematopoietic cell transplantation. Blood.

[9] Bochud P.Y., Chien J.W., Marr K.A., Leisenring W.M., Upton A., Janer M., Rodrigues S.D., Li S., Hansen J.A., Zhao L.P. (2008). Toll-like receptor 4 polymorphisms and aspergillosis in stem-cell transplantation. N. Engl. J. Med..

[10] Sainz J., Lupianez C.B., Segura-Catena J., Vazquez L., Rios R., Oyonarte S., Hemminki K., Forsti A., Jurado M. (2012). Dectin-1 and DC-SIGN polymorphisms associated with invasive pulmonary Aspergillosis infection. PLoS ONE.

[11] Gresnigt M.S., Cunha C., Jaeger M., Goncalves S.M., Malireddi R.K.S., Ammerdorffer A., Lubbers R., Oosting M., Rasid O., Jouvion G. (2018). Genetic deficiency of NOD2 confers resistance to invasive aspergillosis. Nat. Commun..

[12] Cunha D.O., Leao-Cordeiro J.A.B., Paula H., Ataides F.S., Saddi V.A., Vilanova-Costa C., Silva A. (2018). Association between polymorphisms in the genes encoding toll-like receptors and dectin-1 and susceptibility to invasive aspergillosis: A systematic review. Rev. Soc. Bras. Med. Trop..

[13] Cunha C., Di Ianni M., Bozza S., Giovannini G., Zagarella S., Zelante T., D’Angelo C., Pierini A., Pitzurra L., Falzetti F. (2010). Dectin-1 Y238X polymorphism associates with susceptibility to invasive aspergillosis in hematopoietic transplantation through impairment of both recipient- and donor-dependent mechanisms of antifungal immunity. Blood.

[14] Stappers M.H.T., Clark A.E., Aimanianda V., Bidula S., Reid D.M., Asamaphan P., Hardison S.E., Dambuza I.M., Valsecchi I., Kerscher B. (2018). Recognition of DHN-melanin by a C-type lectin receptor is required for immunity to Aspergillus. Nature.

[15] Sainz J., Perez E., Gomez-Lopera S., Jurado M. (2008). IL1 gene cluster polymorphisms and its haplotypes may predict the risk to develop invasive pulmonary aspergillosis and modulate C-reactive protein level. J. Clin. Immunol..

[16] Sainz J., Perez E., Gomez-Lopera S., Lopez-Fernandez E., Moratalla L., Oyonarte S., Jurado M. (2008). Genetic variants of IL6 gene promoter influence on C-reactive protein levels but are not associated with susceptibility to invasive pulmonary aspergillosis in haematological patients. Cytokine.

[17] Cunha C., Goncalves S.M., Duarte-Oliveira C., Leite L., Lagrou K., Marques A., Lupianez C.B., Mesquita I., Gaifem J., Barbosa A.M. (2017). IL-10 overexpression predisposes to invasive aspergillosis by suppressing antifungal immunity. J. Allergy Clin. Immunol..

[18] Mezger M., Steffens M., Beyer M., Manger C., Eberle J., Toliat M.R., Wienker T.F., Ljungman P., Hebart H., Dornbusch H.J. (2008). Polymorphisms in the chemokine (C-X-C motif) ligand 10 are associated with invasive aspergillosis after allogeneic stem-cell transplantation and influence CXCL10 expression in monocyte-derived dendritic cells. Blood.

[19] Cunha C., Carvalho A. (2019). Genetic defects in fungal recognition and susceptibility to invasive pulmonary aspergillosis. Med. Mycol..

[20] Netea M.G., Wijmenga C., O’Neill L.A. (2012). Genetic variation in Toll-like receptors and disease susceptibility. Nat. Immunol..

[21] Wojtowicz A., Bochud P.Y. (2015). Host genetics of invasive Aspergillus and Candida infections. Semin. Immunopathol..

[22] Lupianez C.B., Martinez-Bueno M., Sanchez-Maldonado J.M., Badiola J., Cunha C., Springer J., Lackner M., Segura-Catena J., Canet L.M., Alcazar-Fuoli L. (2020). Polymorphisms within the ARNT2 and CX3CR1 Genes Are Associated with the Risk of Developing Invasive Aspergillosis. Infect. Immun..

[23] Sainz J., Salas-Alvarado I., Lopez-Fernandez E., Olmedo C., Comino A., Garcia F., Blanco A., Gomez-Lopera S., Oyonarte S., Bueno P. (2010). TNFR1 mRNA expression level and TNFR1 gene polymorphisms are predictive markers for susceptibility to develop invasive pulmonary aspergillosis. Int. J. Immunopathol. Pharmacol..

[24] Lupianez C.B., Villaescusa M.T., Carvalho A., Springer J., Lackner M., Sanchez-Maldonado J.M., Canet L.M., Cunha C., Segura-Catena J., Alcazar-Fuoli L. (2016). Common Genetic Polymorphisms within NFkappaB-Related Genes and the Risk of Developing Invasive Aspergillosis. Front. Microbiol..

[25] Cunha C., Giovannini G., Pierini A., Bell A.S., Sorci G., Riuzzi F., Donato R., Rodrigues F., Velardi A., Aversa F. (2011). Genetically-determined hyperfunction of the S100B/RAGE axis is a risk factor for aspergillosis in stem cell transplant recipients. PLoS ONE.

[26] Napolioni V., Pariano M., Borghi M., Oikonomou V., Galosi C., De Luca A., Stincardini C., Vacca C., Renga G., Lucidi V. (2019). Genetic Polymorphisms Affecting IDO1 or IDO2 Activity Differently Associate With Aspergillosis in Humans. Front. Immunol..

[27] Barrios C.S., Johnson B.D., Henderson J.D., Fink J.N., Kelly K.J., Kurup V.P. (2005). The costimulatory molecules CD80, CD86 and OX40L are up-regulated in Aspergillus fumigatus sensitized mice. Clin. Exp. Immunol..

[28] Chiang L.Y., Sheppard D.C., Gravelat F.N., Patterson T.F., Filler S.G. (2008). Aspergillus fumigatus stimulates leukocyte adhesion molecules and cytokine production by endothelial cells in vitro and during invasive pulmonary disease. Infect. Immun..

[29] Sun L., Chen C., Wu J., Dai C., Wu X. (2018). TSLP-activated dendritic cells induce T helper type 2 inflammation in Aspergillus fumigatus keratitis. Exp. Eye Res..

[30] Menon M.B., Gaestel M. (2018). MK2-TNF-Signaling Comes Full Circle. Trends Biochem. Sci..

[31] Wu Y., He H., Ding Y., Liu S., Zhang D., Wang J., Jiang H., Zhang D., Sun L., Ye R.D. (2018). MK2 mediates macrophage activation and acute lung injury by regulating let-7e miRNA. Am. J. Physiol. Lung Cell. Mol. Physiol.

[32] Ronkina N., Shushakova N., Tiedje C., Yakovleva T., Tollenaere M.A.X., Scott A., Batth T.S., Olsen J.V., Helmke A., Bekker-Jensen S.H. (2019). The Role of TTP Phosphorylation in the Regulation of Inflammatory Cytokine Production by MK2/3. J. Immunol..

[33] Lupianez C.B., Canet L.M., Carvalho A., Alcazar-Fuoli L., Springer J., Lackner M., Segura-Catena J., Comino A., Olmedo C., Rios R. (2015). Polymorphisms in Host Immunity-Modulating Genes and Risk of Invasive Aspergillosis: Results from the AspBIOmics Consortium. Infect. Immun..

[34] De Pauw B., Walsh T.J., Donnelly J.P., Stevens D.A., Edwards J.E., Calandra T., Pappas P.G., Maertens J., Lortholary O., Kauffman C.A. (2008). Revised definitions of invasive fungal disease from the European Organization for Research and Treatment of Cancer/Invasive Fungal Infections Cooperative Group and the National Institute of Allergy and Infectious Diseases Mycoses Study Group (EORTC/MSG) Consensus Group. Clin. Infect. Dis..

[35] Gabriel S.B., Schaffner S.F., Nguyen H., Moore J.M., Roy J., Blumenstiel B., Higgins J., DeFelice M., Lochner A., Faggart M. (2002). The structure of haplotype blocks in the human genome. Science.

[36] Vallabhaneni S., Benedict K., Derado G., Mody R.K. (2017). Trends in Hospitalizations Related to Invasive Aspergillosis and Mucormycosis in the United States, 2000–2013. Open Forum. Infect. Dis..

[37] Orru V., Steri M., Sole G., Sidore C., Virdis F., Dei M., Lai S., Zoledziewska M., Busonero F., Mulas A. (2013). Genetic variants regulating immune cell levels in health and disease. Cell.

[38] Aguirre-Gamboa R., Joosten I., Urbano P.C.M., van der Molen R.G., van Rijssen E., van Cranenbroek B., Oosting M., Smeekens S., Jaeger M., Zorro M. (2016). Differential Effects of Environmental and Genetic Factors on T and B Cell Immune Traits. Cell Rep..

[39] Sugamura K., Ishii N., Weinberg A.D. (2004). Therapeutic targeting of the effector T-cell co-stimulatory molecule OX40. Nat. Rev. Immunol..

[40] Murata K., Ishii N., Takano H., Miura S., Ndhlovu L.C., Nose M., Noda T., Sugamura K. (2000). Impairment of antigen-presenting cell function in mice lacking expression of OX40 ligand. J. Exp. Med..

[41] Takeda I., Ine S., Killeen N., Ndhlovu L.C., Murata K., Satomi S., Sugamura K., Ishii N. (2004). Distinct roles for the OX40-OX40 ligand interaction in regulatory and nonregulatory T cells. J. Immunol..

[42] Jenkins S.J., Perona-Wright G., Worsley A.G., Ishii N., MacDonald A.S. (2007). Dendritic cell expression of OX40 ligand acts as a costimulatory, not polarizing, signal for optimal Th2 priming and memory induction in vivo. J. Immunol..

[43] Salek-Ardakani S., Song J., Halteman B.S., Jember A.G., Akiba H., Yagita H., Croft M. (2003). OX40 (CD134) controls memory T helper 2 cells that drive lung inflammation. J. Exp. Med..

[44] Oakley M.S., Majam V., Mahajan B., Gerald N., Anantharaman V., Ward J.M., Faucette L.J., McCutchan T.F., Zheng H., Terabe M. (2009). Pathogenic roles of CD14, galectin-3, and OX40 during experimental cerebral malaria in mice. PLoS ONE.

[45] Duffus K., Lopez-Isac E., Teruel M., Simeon C.P., Carreria P., Ortego-Centeno N., Vicente E., Worthington J., Herrick A.L., Martin J. (2019). Association of TNFSF4 (OX40L) polymorphisms with systemic sclerosis-related calcinosis. Rheumatology.

[46] Deng Y., Tsao B.P. (2010). Genetic susceptibility to systemic lupus erythematosus in the genomic era. Nat. Rev. Rheumatol..

[47] Jindra P.T., Conway S.E., Ricklefs S.M., Porcella S.F., Anzick S.L., Haagenson M., Wang T., Spellman S., Milford E., Kraft P. (2016). Analysis of a Genetic Polymorphism in the Costimulatory Molecule TNFSF4 with Hematopoietic Stem Cell Transplant Outcomes. Biol. Blood Marrow Transplant..

[48] Doherty T.A., Soroosh P., Khorram N., Fukuyama S., Rosenthal P., Cho J.Y., Norris P.S., Choi H., Scheu S., Pfeffer K. (2011). The tumor necrosis factor family member LIGHT is a target for asthmatic airway remodeling. Nat. Med..

[49] Herro R., Da Silva Antunes R., Aguilera A.R., Tamada K., Croft M. (2015). Tumor necrosis factor superfamily 14 (LIGHT) controls thymic stromal lymphopoietin to drive pulmonary fibrosis. J. Allergy Clin. Immunol..

[50] Nguyen N.L., Chen K., McAleer J., Kolls J.K. (2013). Vitamin D regulation of OX40 ligand in immune responses to Aspergillus fumigatus. Infect. Immun..

[51] Kreindler J.L., Steele C., Nguyen N., Chan Y.R., Pilewski J.M., Alcorn J.F., Vyas Y.M., Aujla S.J., Finelli P., Blanchard M. (2010). Vitamin D3 attenuates Th2 responses to Aspergillus fumigatus mounted by CD4+ T cells from cystic fibrosis patients with allergic bronchopulmonary aspergillosis. J. Clin. Investig..

[52] Lee H.C., Ziegler S.F. (2007). Inducible expression of the proallergic cytokine thymic stromal lymphopoietin in airway epithelial cells is controlled by NFkappaB. Proc. Natl. Acad. Sci. USA.

[53] Zhang K., Shan L., Rahman M.S., Unruh H., Halayko A.J., Gounni A.S. (2007). Constitutive and inducible thymic stromal lymphopoietin expression in human airway smooth muscle cells: Role in chronic obstructive pulmonary disease. Am. J. Physiol. Lung Cell. Mol. Physiol..

[54] Kashyap M., Rochman Y., Spolski R., Samsel L., Leonard W.J. (2011). Thymic stromal lymphopoietin is produced by dendritic cells. J. Immunol..

[55] Lee K.H., Cho K.A., Kim J.Y., Kim J.Y., Baek J.H., Woo S.Y., Kim J.W. (2011). Filaggrin knockdown and Toll-like receptor 3 (TLR3) stimulation enhanced the production of thymic stromal lymphopoietin (TSLP) from epidermal layers. Exp. Dermatol..

[56] Ni G., Chen Y., Wu F., Zhu P., Song L. (2017). NOD2 promotes cell proliferation and inflammatory response by mediating expression of TSLP in human airway smooth muscle cells. Cell Immunol..

[57] Qiao J., Li A., Jin X. (2011). TSLP from RSV-stimulated rat airway epithelial cells activates myeloid dendritic cells. Immunol. Cell Biol..

[58] Kato A., Favoreto S., Avila P.C., Schleimer R.P. (2007). TLR3- and Th2 cytokine-dependent production of thymic stromal lymphopoietin in human airway epithelial cells. J. Immunol..

[59] Ito T., Wang Y.H., Duramad O., Hori T., Delespesse G.J., Watanabe N., Qin F.X., Yao Z., Cao W., Liu Y.J. (2005). TSLP-activated dendritic cells induce an inflammatory T helper type 2 cell response through OX40 ligand. J. Exp. Med..

[60] Wang Y.H., Ito T., Wang Y.H., Homey B., Watanabe N., Martin R., Barnes C.J., McIntyre B.W., Gilliet M., Kumar R. (2006). Maintenance and polarization of human TH2 central memory T cells by thymic stromal lymphopoietin-activated dendritic cells. Immunity.

[61] Jiang Q., Su H., Knudsen G., Helms W., Su L. (2006). Delayed functional maturation of natural regulatory T cells in the medulla of postnatal thymus: Role of TSLP. BMC Immunol..

[62] Osborn M.J., Ryan P.L., Kirchhof N., Panoskaltsis-Mortari A., Mortari F., Tudor K.S. (2004). Overexpression of murine TSLP impairs lymphopoiesis and myelopoiesis. Blood.

[63] Redhu N.S., Saleh A., Halayko A.J., Ali A.S., Gounni A.S. (2011). Essential role of NF-kappaB and AP-1 transcription factors in TNF-alpha-induced TSLP expression in human airway smooth muscle cells. Am. J. Physiol. Lung Cell. Mol. Physiol..

[64] Craxton A., Shu G., Graves J.D., Saklatvala J., Krebs E.G., Clark E.A. (1998). p38 MAPK is required for CD40-induced gene expression and proliferation in B lymphocytes. J. Immunol..

[65] Sutherland C.L., Heath A.W., Pelech S.L., Young P.R., Gold M.R. (1996). Differential activation of the ERK, JNK, and p38 mitogen-activated protein kinases by CD40 and the B cell antigen receptor. J. Immunol..

[66] Sreekanth G.P., Chuncharunee A., Sirimontaporn A., Panaampon J., Noisakran S., Yenchitsomanus P.T., Limjindaporn T. (2016). SB203580 Modulates p38 MAPK Signaling and Dengue Virus-Induced Liver Injury by Reducing MAPKAPK2, HSP27, and ATF2 Phosphorylation. PLoS ONE.

[67] Pan W., Zhu S., Dai D., Liu Z., Li D., Li B., Gagliani N., Zheng Y., Tang Y., Weirauch M.T. (2015). MiR-125a targets effector programs to stabilize Treg-mediated immune homeostasis. Nat. Commun..

[68] Swertz M.A., Dijkstra M., Adamusiak T., van der Velde J.K., Kanterakis A., Roos E.T., Lops J., Thorisson G.A., Arends D., Byelas G. (2010). The MOLGENIS toolkit: Rapid prototyping of biosoftware at the push of a button. BMC Bioinform..

[69] Wilkinson M.D., Dumontier M., Aalbersberg I.J., Appleton G., Axton M., Baak A., Blomberg N., Boiten J.W., da Silva Santos L.B., Bourne P.E. (2016). The FAIR Guiding Principles for scientific data management and stewardship. Sci. Data.

