# Supplementary material for: Polymorphisms within the TNFSF4 and MAPKAPK2 Loci Influence the Risk of Developing Invasive Aspergillosis: A Two-Stage Case Control Study in the Context of the aspBIOmics Consortium
Source: J Fungi (Basel). 2020 Dec 23;7(1):4. doi: 10.3390/jof7010004 (PMC7823601; doi:10.3390/jof7010004)
Supplement: Supplementary file 1 [file jof-07-00004-s001.zip › jof-1029381 Supplementary_Table_1_SNP_list.docx]

**Supplementary Table 1.** Selected SNPs within the *TNFSF4* and *MAPKAPK2* loci.

| SNP_rsID | Chr_position (GRCh38) | Gene | Risk allele |
| --- | --- | --- | --- |
| rs10489269 | 1_173190924 | TNFSF4 | A |
| rs7518045 | 1_173194373 | TNFSF4 | G |
| rs10489266 | 1_173209314 | TNFSF4 | G |
| rs2205959 | 1_173222232 | TNFSF4 | G |
| rs61828280 | 1_173301595 | TNFSF4 | G |
| rs4916320 | 1_173314948 | TNFSF4 | G |
| rs4357565 | 1_173330094 | TNFSF4 | C |
| rs1342038 | 1_173332377 | TNFSF4 | A |
| rs947505 | 1_173332958 | TNFSF4 | A |
| rs56307807 | 1_173356076 | TNFSF4 | C |
| rs6425219 | 1_173371277 | TNFSF4 | T |
| rs1418191 | 1_173393318 | TNFSF4 | C |
| rs1578624 | 1_173399532 | TNFSF4 | C |
| rs7526628 | 1_173407740 | TNFSF4 | T |
| rs7549074 | 1_173445045 | TNFSF4 | C |
| rs6700269 | 1_173455483 | TNFSF4 | T |
| rs12140760 | 1_173460856 | TNFSF4 | A |
| rs17013271 | 1_206637601 | DYRK3 | T |
| rs6540512 | 1_206640392 | DYRK3 | A |
| rs12126682 | 1_206643926 | DYRK3 | G |
| rs4845123 | 1_206647192 | DYRK3 | A |
| rs17435120 | 1_206661832 | DYRK3\|MAPKAPK2 | G |
| rs12407425 | 1_206662276 | DYRK3\|MAPKAPK2 | A |
| rs11119267 | 1_206667544 | DYRK3\|MAPKAPK2 | C |
| rs12038489 | 1_206671625 | DYRK3\|MAPKAPK2 | A |
| rs61815610 | 1_206693042 | MAPKAPK2 | T |
| rs10863788 | 1_206703461 | MAPKAPK2 | A |
| rs12123706 | 1_206703530 | MAPKAPK2 | T |
| rs4072677 | 1_206705777 | MAPKAPK2 | G |
| rs61815626 | 1_206708825 | MAPKAPK2 | A |
| rs12030124 | 1_206710508 | MAPKAPK2 | T |
| rs4256810 | 1_206717104 | MAPKAPK2 | C |
| rs28394820 | 1_206722910 | MAPKAPK2 | C |
| rs4073250 | 1_206731698 | MAPKAPK2 | T |
| rs7515374 | 1_206734678 | MAPKAPK2\|\|IL10 | C |
| rs12137965 | 1_206737835 | MAPKAPK2\|\|IL10 | G |

Abbreviations: SNP, single nucleotide polymorphism; MAPKAPK2, MAPK activated protein kinase 2; DYRK3, dual specificity tyrosine phosphorylation regulated kinase 3; IL10, interleukin-10;
